# Application of The Consolidated Framework for Implementation Research to inform understanding of barriers and facilitators to the implementation of opioid and naloxone training on college campuses

**DOI:** 10.1186/s43058-023-00438-y

**Published:** 2023-05-23

**Authors:** Rachel C. Shelton, Kathleen Goodwin, Michael McNeil, Melanie Bernitz, Savannah P. Alexander, Carrigan Parish, Laura Brotzman, Matthew Lee, WaiKwan (Bonnie) Li, Supriya Makam, Nicholas Ganek, Dean Foskett, Chloe Warren, Lisa R. Metsch

**Affiliations:** 1 Department of Sociomedical Sciences, Columbia University Mailman School of Public Health, Columbia University, 722 W 168th St, Room 941, New York, NY 10032 USA; 2 Columbia University Vagelos College of Physicians and Surgeons, New York, NY USA; 3 Columbia Health, Columbia University, New York, NY USA; 4 Center for Family and Community Medicine, Columbia University Vagelos College of Physicians and Surgeons, New York, NY USA; 5 Columbia University, New York, NY USA; 6 School of General Studies, Columbia University, New York, NY USA

**Keywords:** Theory, Frameworks, College campus, Implementation, Naloxone, Opioid

## Abstract

**Background:**

The opioid epidemic in the US continues to worsen. Opioid-only and polysubstance-involved opioid overdose deaths are increasing among adolescents and young adults, who have limited knowledge of opioid overdose prevention, including recognition and response. College campuses have infrastructure to support national-level implementation of evidence-based public health strategies for providing opioid overdose prevention and naloxone training programs among this priority population. However, college campuses are an underutilized, understudied setting for this programming. To address this gap, we conducted research assessing barriers and facilitators to planning and implementing this programming on college campuses.

**Methods:**

We held 9 focus groups among purposively selected campus stakeholders whose perceptions were important to understand in planning for the dissemination and implementation of opioid overdose prevention and naloxone training. Focus group scripts were informed by The Consolidated Framework for Implementation Research (CFIR) to query about perceptions of opioid and other substance use, opioid and other substance use-related resources, and naloxone administration training. We used a deductive-inductive, iterative approach to thematic analysis.

**Results:**

Themes about implementation barriers included (1) the perception that problematic use of other (non-opioid) substances was more prevalent than opioid use on campus and focus on those substances would be a greater priority on college campuses; (2) student schedules were overwhelmed with academic commitments and extracurricular activities, making delivery of additional training challenging; (3) barriers related to the perceived complexity and decentralization of communication on campus, preventing students from knowing how to access substance use-related resources. Themes about implementation facilitators included (1) framing naloxone training as important in becoming a responsible leader on campus and in the broader community and (2) leveraging existing infrastructure, champions within existing campus groups, and tailored messaging to facilitate participation in naloxone training.

**Conclusions:**

This is the first study to provide in-depth insights into potential barriers and facilitators to widespread, routine implementation of naloxone/opioid education on undergraduate college campuses. The study captured diverse stakeholder perspectives and was theoretically grounded in CFIR, contributing to the growing literature on the application and refinement of CFIR across diverse community and school contexts.

**Supplementary Information:**

The online version contains supplementary material available at 10.1186/s43058-023-00438-y.

Contributions to the literature

- The Consolidated Framework for Implementation Research (CFIR) has mostly been applied in healthcare contexts, but we need to understand how to apply CFIR in diverse community contexts where evidence-based public health programs are needed.
- Our study provides insight into relevant CFIR domains and constructs in the context of delivering naloxone and opioid education on undergraduate college campuses.
- Evaluation of multi-level contextual factors and barriers and facilitators to implementation on an undergraduate college campus can inform future research that aims to advance understanding of widespread dissemination and implementation of naloxone training and other evidence-based public health programs in this understudied setting.

## Background

The United States (US) is experiencing a national epidemic of opioid- and stimulant-use and overdose-related deaths. The situation has worsened with the dramatic rise in the availability and use of opioids, particularly synthetic opioids (primarily illicitly manufactured fentanyl (IMF)) [1, 2]. Based on the most recent provisional data from the Centers for Disease Control and Prevention (CDC), the number of drug overdose deaths that occurred during the year ending in April 2022 (108,174) is over 6 times the number that occurred in 1999 (16,849) [3, 4]. Seventy-six percent (81,692) of those 108,174 deaths involved opioids, 89% of which involved synthetic opioids [4]. City, county, state, and national-level data support that the COVID-19 pandemic exacerbated (or at least maintained) the pre-existing upward trend of opioid-related overdoses and resultant deaths [4–10], with the number of opioid-related and synthetic opioid-related overdose deaths increasing by 52% and 79%, respectively, between the years ending in March 2020 and in April 2022 [4]. A number of factors likely contributed to this exacerbation, including social isolation, job loss, worsening mental health, and lack of widespread access to mental healthcare, opioid use disorder (OUD) treatment, and harm reduction services [2, 5, 8, 10–15].

Young adults are a priority population in the opioid overdose epidemic [16–18]. College-aged adults [18–25] are more likely than other age groups to misuse opioids generally (e.g., prescription pain reliever misuse or heroin use) and IMF specifically, and have worse OUD treatment outcomes (e.g., higher rates of 24-week relapse than older adults) [13, 19, 20]. Based on national CDC data, between 1999 and 2018 opioid-only and polysubstance-involved opioid overdose deaths (primarily involving synthetic opioids and cocaine) among adolescents and young adults ages 13–25 increased by 384% and 760%, respectively [21]. After remaining consistent from 2010 through 2019, the number of drug overdose deaths among adolescents ages 14–18 increased from 492 (2.36 per 100,000) to 954 (4.57 per 100,000) between 2019 and 2020 and rose to 1146 deaths (5.49 per 100,000) in 2021, 77% of which involved fentanyl [22].

Among college students in particular, prescription opioid misuse (e.g., “use without a medical prescription or the use for something other than directed by a prescribing healthcare provider” [23]) is associated with suicidality, depression, anxiety, other forms of psychological distress, and other substance use [23–27], and illicit opioid use (e.g., heroin) is associated with relationship problems like intimate partner violence [24]. A recently published systematic review (2013–2019) found that lifetime prescription opioid misuse prevalence among college students in the US ranged from 4 to 19.7% [23]. Recent data from the American College Health Association-National College Health Assessment (*n* = 90,503 students across 162 colleges) found that 4.8% of college students reported prescription opioid misuse within the past year [28]. Importantly, research indicates that college students have limited knowledge about what constitutes an opioid (e.g., lack of recognition of fentanyl), opioid overdose causes, opioid overdose and withdrawal signs and symptoms, and the importance of naloxone as an opioid antagonist to reverse opioid overdose [29]. Similarly, research supports that there is a low perceived risk of opioid overdose death among adolescents and young adults, who may not change use patterns even after personally experiencing an overdose [30].

Naloxone training programs that provide information about opioid overdose prevention, recognition, and response have shown effectiveness across a range of settings in improving overdose knowledge and response skills and reducing stigma (e.g., see Razaghizad and colleagues’ recent umbrella review) [31]. Additionally, such programs have documented successful overdose reversals involving peer-administered naloxone, few adverse consequences, and reductions in population-level opioid-related mortality [31]. There is growing evidence of the value of community-based distribution of naloxone to laypersons and recent legislation and national efforts to expand naloxone access [32, 33].

College campuses are an important but underutilized, understudied setting where implementation of evidence-based public health strategies for providing education and training around opioid use and naloxone are needed, particularly given the substantial reach (of young adults) and infrastructure they provide for large-scale implementation efforts nationally. Training students and staff/faculty may prevent overdoses not only on campus, but in surrounding communities, as well as in communities to which students go home during breaks and after graduating [34]. Such training fills a critical need and is part of a broader, multi-pronged public health approach, as many opioid overdose deaths occur in the presence of bystanders who are not prepared to respond [1, 35, 36].

In recent years, the American College Health Association Guidelines “Opioid Prescribing in College Health” have recommended stocking naloxone and training health center staff about its use [37]. Nationally, universities have differed in their perspective about naloxone, specifically regarding who should be trained and what protocols established for overdose prevention and response. To date, opioid overdose prevention and naloxone training programs have primarily been offered to medical, pharmacy, and undergraduate/graduate nursing students [38–52], with few examples implemented for undergraduate students generally [34, 53–56]. As one example, in 2016, the University of Texas at Austin Schools of Pharmacy and Social Work launched Operation Naloxone, an interprofessional collaboration between students and faculty aimed at combating the opioid overdose crisis through a multi-pronged approach that addressed knowledge gaps in substance use safety and overdose prevention and ensured that communities were prepared with naloxone and other resources to prevent overdoses and overdose deaths. This approach included a service learning component for all students on campus [54]. University of Southern California implemented a program modeled after Operation Naloxone and is offering training online to improve accessibility during the pandemic [55]. Emerging literature suggests that online naloxone training may be as effective as in-person training (e.g., in knowledge improvement) [38, 39, 52]. Importantly, though there are several examples of training programs emerging in the gray literature, there are significant gaps in routine delivery and evaluation of implementation of such training on college campuses nationally [53, 55, 57–60].

**Fig. 1:**
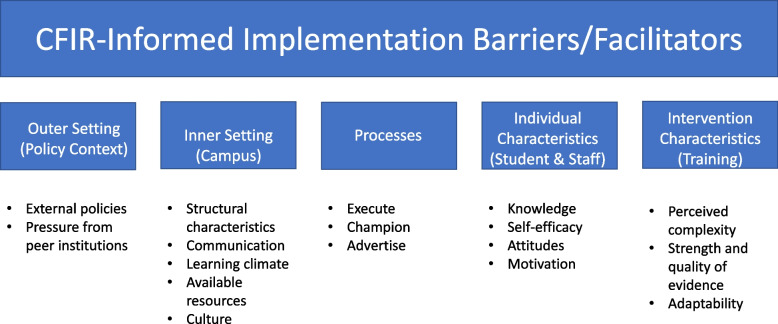
Relevant CFIR constructs, organized by CFIR domain, that guided qualitative data collection and analysis

As evidence of the importance and impact of naloxone programs continues to grow as an essential part of overdose prevention efforts [53, 61], it is important to understand challenges and facilitators to planning and implementing such programs on college campuses nationally. To address these gaps and understand the potential acceptability and feasibility of implementation in this understudied setting, we conducted research to assess key barriers and facilitators to the delivery of opioid overdose prevention and naloxone training programs on college campuses. Specifically, we use an implementation science framework (The Consolidated Framework for Implementation Research (CFIR) [62]; see Fig. 1) to inform qualitative data collection and analysis among students and staff on Columbia University’s undergraduate campus. This paper seeks to help advance the understanding of multi-level factors that may impact the routine, widespread delivery of naloxone and opioid education and training on college campuses.

## Methods

### Recruitment and data collection

We conducted a literature review and environmental scan (e.g., of relevant programs and trainings nationally and on Columbia University’s undergraduate campus) to assess existing resources and initiatives and help identify key stakeholder groups whose perceptions were important to understand in planning for the dissemination and implementation of training on overdose prevention and naloxone administration. Key stakeholders were identified in collaboration with campus partners, including university student life, student veterans, student health service (Columbia Health), and an ongoing taskforce focused on substance use prevention and treatment that was part of a larger effort to enhance mental health service delivery on Columbia’s undergraduate campus. Previous research had suggested that students who identify as male, white, members of social fraternity and sorority houses and off-campus houses, have lower grade-point averages, and attend more competitive colleges may be priority groups for naloxone training/opioid education on college campuses [63–65]. Informed by the campus environmental scan and literature review, we sought to identify student groups that may have members at elevated risk for opioid use or may be well-positioned to disseminate opioid use-related resources or intervene in an overdose situation. Groups included resident assistants (RAs), fraternities, sororities, military veterans, campus recovery coalition members, and student athletes. To complement and expand upon student focus groups, we included a focus group to capture Columbia Health staff/administrator perceptions (staff that lead health-related campus programming).

Purposeful sampling enhanced the representation of these key sub-groups. Nine focus groups were conducted between March 2019 and October 2019. Focus group scripts queried about perceptions of opioid and other substance use on campus, opioid and other substance use-related resources, and naloxone administration training. Focus groups employed semi-structured questions to explore perceived needs in substance use education and training and examined potential barriers and facilitators to receiving training and administering naloxone on campus and in students’ local communities. Data collection was broadly informed by questions related to the five key CFIR domains; for the guide, we used probes that aligned and mapped onto CFIR domains as we sought to understand barriers/facilitators at each level (e.g., Outer/policy Setting, Inner/campus Setting, Processes, Individual (Staff/Student) Characteristics, Intervention Characteristics) [62]. We selected CFIR because it is theoretically and empirically grounded and provides a strong foundation for conducting a comprehensive, multi-level assessment in planning for implementation. Focus groups were conducted by research assistants with training and experience in qualitative data collection and opioid/substance use research (see Additional file 1). All nine focus groups were audio-recorded and transcribed. A total of 60 key stakeholders participated (7 staff, 53 students). Student focus groups consisted of three groups of fraternity members and one group each of sorority members, student athletes, recovery coalition members, military veterans, and RAs (5–11 participants per focus group). Students from all four Columbia University undergraduate schools (Columbia College, Columbia School of Engineering and Applied Sciences, Columbia School of General Studies, Barnard College) participated. We compensated each participant for their time with a $20 gift card.

### Data analysis

Two coders (RCS, KG) used an iterative approach to coding and thematic analysis. Coders used a deductive-inductive coding process, whereby they initially coded two focus group transcripts using initial categories informed by the focus group guide and key CFIR domains and constructs, and then inductively identified new categories to add to the codebook. All nine transcripts were coded independently by both coders using the full codebook. For the “Outer setting” CFIR domain, we coded factors related to off-campus/New York City (NYC) local communities, the national opioid epidemic, and laws/policies. The “Inner setting” reflected qualities specific to Columbia University students and administration, including university-wide communication, attitudes about naloxone/opioid training implementation, and campus opioid and other substance use-related resources. For “Processes,” we focused on perceptions of naloxone administration and overdose prevention training, including how to communicate about, recruit for, and deliver trainings to students/staff on campus. “Individual characteristics” (students, staff) included perceptions and attitudes about substance use, as well as opinions and knowledge specific to opioid use, the opioid epidemic, and naloxone (both support for implementing naloxone trainings and concerns about using naloxone once trained). For “Intervention characteristics,” we considered elements regarding the naloxone/opioid trainings including scheduling, content, and factors facilitating widespread student participation. Focus groups continued until we reached saturation. We abided by the Consolidated Criteria for Reporting Qualitative Research (see Additional file 2).

## Results

### Sample characteristics

Sociodemographic characteristics of student focus group participants are summarized in Table 1. Over half of participating students (53%) identified as female and 68% as white, 11% as Asian, and 8% as Black or African American. Student participants ranged in age from 18 to 42 (mean age: 22). An additional focus group was conducted with 5 female and 2 male Columbia Health staff members representing clinicians, health promotion specialists and staff from violence prevention/response teams. Staff participants ranged in age from 27 to 49 (mean age: 34).

**Table 1:** Sociodemographic characteristics of student focus groups

Student demographics (*N* = 53)	
**Gender**	
Female	53% (28)
Male	47% (25)
**Race**	
White	68% (36)
Black or African American	8% (4)
Asian	11% (6)
Multiracial	8% (4)
Other	6% (3)
**Ethnicity**	
Hispanic	12% (4)
Not Hispanic	88% (49)
**Age range**	18—42
**Age mean**	22
**Student year**	
Freshman	11% (6)
Sophomore	26% (14)
Junior	26% (14)
Senior	36% (19)
**Student school**	
Columbia College	64% (34)
Columbia School of Engineering and Applied Sciences	6% (3)
Columbia School of General Studies	25% (13)
Barnard	6% (3)
**Focus group**	
Fraternities	36% (19)
Military Veterans	9% (5)
Resident Assistants	9% (5)
Recovery Coalition	9% (5)
Sororities	15% (8)
Student Athletes	21% (11)

### Overview of key overarching themes

Themes were organized according to the five CFIR domains; we summarized overarching themes related to student and staff-identified barriers and facilitators to dissemination and implementation of opioid overdose prevention and naloxone trainings for Columbia University undergraduate students. There were several overarching themes related to barriers to routine implementation, including (1) the perception that problematic use of other (non-opioid) substances (e.g., alcohol, marijuana) was more prevalent than opioid use on campus and focus on those substances would be a greater priority on campus; (2) student schedules were overwhelmed with academic commitments and extracurricular activities, making the delivery of additional training challenging; (3) barriers related to the perceived complexity and decentralization of campus communication, preventing students from easily knowing how to access substance use-related resources. Overarching themes related to important facilitators of routine, widespread uptake on campus included (1) framing naloxone training as important in becoming a responsible leader on campus and in the broader community (e.g., being a global citizen) and (2) partnering and leveraging existing infrastructure and champions within campus or social groups (e.g., RAs, fraternities/sororities) to facilitate and incentivize participation in naloxone training, including using tailored strategies and messaging to reach specific groups. In Table 2, we summarize barriers to and recommended strategies for facilitating implementation of naloxone/opioid training that are relevant to specific student groups. Below and in Tables 3 and 4, we summarize key barriers and facilitators we identified within the five CFIR domains, which are italicized when they relate to specific CFIR constructs.

**Table 2:** Barriers to and recommended strategies for facilitating the implementation of naloxone/opioid training for priority student groups

Group affiliation	Barriers	Recommendations
***Fraternity and Sorority members***	Required to attend additional trainings for Greek life requirements causing increased training fatigue among members	Incentivize by integrating into and aligning with Greek life requirement
***Student Athletes***	Risk of opioid prescription after surgery and stigma around use	Have athletic trainers/ coaches (existing internal “program champions”) communicate about trainings
***Student Recovery Coalition members***	Messaging about naloxone may conflict with 12-step community programs	Have professors highlight opportunities for trainings and offer extra credit for participation
***Student Military Veterans***	Disconnected from campus communications due to being non-traditional students	Use social media to disseminate information about substance use resources
***Columbia Health Staff members***		Appoint or invite “health ambassadors” (students interested in health professions) to participate in trainings
***Resident Assistants***	Perceived as mandatory reporters, which may result in students avoiding disclosing opioid use to them

**Table 3:** Key barriers to implementation of naloxone training and exemplar quotes organized by CFIR Domains

Domains	Key barriers	Exemplar quotes
Outer setting	- Stigma surrounding disclosing opioid use and seeking help for any substance use disorder - Legal repercussions of opioid use and lack of clarification around campus policies and laws	“I think there’s a stigma that exists for students going through this problem [addiction] and it’d be hard, I think, for many students to first admit to the problem, acknowledge that it exists and then actively go and seek help for a problem they’re currently dealing with and I think that of the personal road block for individuals what I think would be the number one factor in them pursuing help or not pursuing help”—Fraternity member
Inner setting	- Decentralized student body makes communication challenging - Competing university-wide initiatives and priorities (sexual assault prevention, higher-risk drinking prevention, etc.) and training fatigue - Insufficient staffing and advertising for student mental health and substance use treatment - Perceived lack of widespread knowledge about opioid use and lack of prioritization	“Sophomore year I had surgery and there really was just like a coach or two that would come up to me and say – so you’re off the painkillers now, right?…But in terms of an open supportive culture, or any mechanisms that are in place, like not really.”—Student athlete “If you go to Columbia websites, there’s no health resources for students who are struggling. That’s not something that’s advertised…it’s never front page news. It’s always like way in the back in the classifieds.”—Recovery Coalition member
Processes	- Complexity and decentralization of University’s structure and communication - Difficult to disseminate messages broadly to reach such a large and diverse student population	“Especially on a campus that’s this large and it really is decentralized.”—Recovery Coalition member
Individual characteristics	- Opioid use not perceived as widespread problem on campus - Limited knowledge about naloxone and risk of overdose with drug mixing - Lack of perceived opioid use among students/ stigma specific to opioids - Concern about assuming responsibility for others’ lives/inappropriate or inexpert use of naloxone (low self-efficacy) - Fear of mandatory reporting and academic consequences	“I would say that would be my one number one biggest distrust is [not] knowing when they have to report it and when they don’t.”—Student athlete “…if you wanted to ask your RA a question, like is this dangerous or I need help with this, I feel like there’s a lot of hesitation 'cause you don’t know if they have to like report that to someone like people are just scared to be, to get in trouble too so there’s this like lack of knowledge of who you can trust and like who you can ask for like those resources”—Sorority member “…for RAs [the] role is kind of like how do you like get trust from people based on like you can’t keep anything like confidential?”—Resident Assistant “If somebody on campus is using drugs because they’re stressed out and maybe they just take pills to go through the day or get their homework done but when they’re not expecting it, that might be, might be the overdose, might just be…the end.”—Recovery Coalition member
Intervention characteristics	- Competing demands for students’ time due to rigorous academic and extracurricular responsibilities - Concern that increasing naloxone access may encourage opioid use - Limited access to kits in buildings and ability to carry kits on person - Difficulty identifying students with greatest need for training	“I mean people are very busy, finding the time to like do anything at Columbia is kind of difficult…”—Fraternity member “…regardless of what’s done parents usually have an adverse reaction…they’re like ‘my children just study,’ ‘my children don’t drink, they don’t do drugs, they don’t do this, this isn’t gonna happen to them’ and by you talking about it you’re promoting that idea.”—Columbia Health staff member “Administrators might look at it at first from an issue of liability and you have to get through the liability concerns.”—Military veteran

**Table 4:** Key facilitators to implementation of naloxone training and exemplar quotes organized by CFIR domain

Domains	Key facilitators/recommendations	Exemplar quotes
Outer setting	- Students have awareness of opioid epidemic affecting wider NYC community - Address opioid use-related stigma in trainings - Administration feels pressure to initiate trainings if peer institutions are doing the same or wants to be seen as leader - Clarify risk of legal repercussions and mandatory reporters	“We are in a city where these drugs are very prevalent and I feel like there’s definitely a possibility of us coming across someone who ODs here, and wherever we go after college.”—Sorority member “For example… in the state of New York there’s legislation that there’s an amnesty clause…so if it’s part of administering this [naloxone] that there’s not necessarily going to be a punishment off of that, there might be like basics or counseling or other things but it’s not necessarily a sanction um that can also be helpful… 'cause those questions are gonna come up from students”—Columbia Health staff member
Inner setting	- Simplify and standardize how to access mental health and substance use resources - Centralize campus communication - Promote positive norms around seeking mental health and substance use resources	“I think that we need to, in a way, work on all this on campus, to bring the subject to the table in which we can begin to tackle the stigma part of it. (someone: “yeah”) Um and make more of a normal conversation, something that can be easily discussed but there there’s uh somebody, a speaker that comes and talks about uh the epidemic or um addiction or just make it known, available that there’s resources or maybe there’s a safe place where they can go and talk about it”—Recovery Coalition member
Processes	- Integrate and align incentives within existing structures (e.g., Greek life requirements, extra credit in courses) - Frame trainings as important to NYC community and demonstrating leadership - Advertise trainings in frequently accessed buildings and through social groups (i.e., fraternities and sororities) - Engage campus leaders: **Fraternity members** recommend fellow fraternity members **Student athletes** recommend trainers and coaches **Recovery coalition members** recommend professors and senior students **Health staff members** recommend choosing specific groups to complete training first to generate “buzz” then disseminate widely	“what if you strategically pick certain groups and like offer it to them first as sort of like, you beta, like, selling the training and then you know that generates maybe like its own little buzz like it kind of has its own thing and then there’s more maybe in the next new student orientation period”—Columbia Health Staff member It’s also like, we get these big emails with a whole bunch of things and a lot of like, they’re very well designed, but there’s so much information I don’t really care about. Whereas like sometimes like it might take more effort but if I get like a personal email from somebody with like a little yellow arrow, I’ll probably read that and if somebody looks personal, they’re like – ‘Hey, naloxone training, come through’” – I might be more inclined to go to that.—Fraternity member
Individual characteristics	- Students and administrators generally have progressive attitudes towards naloxone use - Perceived widespread buy-in among students/ administrators to prevent opioid overdose - Students recognize that opioid use is a problem “back home” in the cities and states that they grew up in - Empower students and specific student groups to promote safety among peers and community - Include recognizing overdose and appropriate opportunities to intervene in trainings	“I think there’s no reason why people shouldn’t be trained in it… If there was one case in my four years that I would have been able to use it and save someone’s life I would have been more than happy to have done that training during NSOP [first-year orientation] and carried around the kit necessary to do that”—Fraternity member “I tend to believe that if the message is about like civil responsibility like help your neighbor people would like be responsive … at least for the good of the greater society and we all need to rely on each other at certain times so like therefore you should know about this, I think that’s like that’s a very positive message and it's also … it doesn’t uh create this like segregation of like “there’s drug users, there’s people…” it’s just like more positive like attitude towards this”—Resident Assistant
Intervention characteristics	- Trainings can be integrated into first-year orientation, broader substance use trainings, and life-saving trainings (e.g., CPR) - Extracurricular and social group affiliations can disseminate information to reach specific groups - Educate students about the opioid epidemic in NYC and at the national level to raise awareness of problem - Widely advertise opportunities to attend trainings through multiple media channels (posters, emails, etc.) - Tailor trainings to needs and concerns of students - Incentivize participation; make trainings in-person and convenient	“I think the first thing that you got to do to make any initiative successful is make it more salient and raise people’s attention”—Military veteran “…it falls on deaf ears 'cause they don’t even think it’s real so you got to find data, you got to find information, you got to bring people together, you got to start having conversations, town halls, make it more salient, create the information, put a report together, tell people, look, this is an issue and then they have to address it”—Military veteran “…it’d be cool if we get to actually see the use rate on campus and in our area of New York City and then the broader city as well. 'Cause, like it’s hard to sit here and discuss what would work like the best, because I don’t know the underlining [underlying] issue that well”—Fraternity member

### Outer setting

#### Barriers

Participants commonly spoke about significant challenges related to societal substance use stigma, with many noting that opioid use carries more stigma than other substance use, e.g., marijuana or alcohol use. Most participants expressed it is taboo and stigmatized to discuss using opioids and they know of very few students on campus who openly admit to using opioids. According to one RA:

...there’s definitely a strong stigma...A marijuana user is not necessarily a bad person or not necessarily a failure, not necessarily a loser or you know, a lot of those negative connotations that people have for someone who is an opioid abuser

Many students also expressed concerns about campus-level and/or broader legal repercussions of recreational opioid use, and confusion about punitive policies that may impact them if they administered naloxone to someone who was overdosing. Health staff members echoed that the primary cause of student apprehension would be about getting themselves or another student in trouble if they administered naloxone to a student that was overdosing. Some students also said they have hesitated to call emergency medical services because they fear they or the student who is using substances will suffer consequences from the University (i.e., expulsion from university housing, requirement to meet with administrators). Other students reported being aware that campus emergency medical services have an amnesty policy that protects students from repercussions for calling emergency medical services for substance use-related cases, but reported this *policy* is neither universally known nor trusted by students who are aware of it.

#### Facilitators/recommendations

All participants noted that students on campus are aware of the opioid epidemic’s harmful impact on the broader NYC community and are therefore motivated to learn more about opioid overdose and how to prevent it. Several students recommended directly addressing opioid-related stigma in naloxone trainings and clarifying *external policies* that would apply to instances when they may use naloxone, including amnesty/Good Samaritan laws^1^. Health staff also noted that the university is highly attentive to other universities’ substance use initiatives and is potentially subject to *pressure from peer* institutions. They believe campus administrators might be more motivated to provide naloxone trainings if they would be viewed as a leader among universities or if other universities had already begun trainings (e.g., an established social norm among peer institutions).

### Inner setting

#### Barriers

Many student participants spoke about *structural characteristics* of universities that may impede the widespread adoption of naloxone training. For example, the diversity and large number of students on campus can make it challenging to reach all students (or those most at risk) with compelling messaging. Such efforts may be further impeded by the decentralization of the undergraduate student body into four schools. Some students perceived that these characteristics can create siloes and barriers that make it difficult to successfully *communicate* and reach the entire student body effectively and efficiently with “one-size-fits-all” messaging that meets the needs and concerns of all student groups.

Most staff and student participants did not perceive opioid use as a priority concern for students or campus administrators, in contrast to recent initiatives on sexual assault and higher-risk drinking prevention, which affect most students and have resulted in the use of university resources and many required student trainings. Regarding *learning climate*, most participants worried that students already experience training fatigue, which would lessen enthusiasm for participating in another training.

In terms of *available resources*, participants differed in their views. Health staff members (providers of mental health and substance use resources) largely felt that there are many services available to students, but that many students are not taking advantage of them. In contrast, students spoke about a *culture* where their peers were reluctant to seek help related to mental health or substance use because it would imply weakness or failure. According to one RA:

...especially here, people are really independent and think they can do things by themselves most of the time, but you have a problem, sounds like it would be hard to acknowledge that you need help with it.

Some students also spoke about perceptions of limited campus resources, including counseling for substance use disorders, due to insufficient staffing and appointment availability. Others commented that there are many substance use-related campus resources, but they are not widely known or advertised, and are difficult for students to access and navigate.

#### Facilitators/recommendations

Both students and staff emphasized that campus communication about naloxone trainings and related substance use resources should be centralized to reach all students regardless of school affiliation. Participants suggested that leadership/administration promote positive norms around seeking help for mental health and substance use issues. Such an effort could facilitate *culture* change and supportive social norms regarding destigmatizing mental health and substance use (and use of related services) on campus. Almost all student and staff participants agreed that mental health and substance use-related resources should be clearly advertised and access should be simple and standardized. According to a Recovery Coalition member:

I also think it’s like there are existing programs that are really good, and really effective, there are a ton of different administrative arms of Columbia Health with fully staffed, with wonderful people, that are working really hard, and students just sort of aren’t aware that they exist.

### Processes

#### Barriers

Many student participants emphasized that the university’s large size and decentralized nature (e.g., separation of students into schools, varying membership in student organizations) may impede implementation, including broad access to communication needed to widely *execute* naloxone trainings. One Military Veteran shared:

Our University in general is so decentralized, there’s so many random disparate groups and organizations and clubs, I’m sure you’ve encountered those difficulties... it’s hard sometimes to find where there’s like one united front addressing an issue and sometimes... they don’t even know that the others exist.

#### Facilitators/recommendations

Many students perceived that tailored messages *championed* by specific student groups or peer leaders would be effective to facilitate the adoption and support the training. Participants suggested that naloxone trainings could also be incorporated into existing campus organization incentive structures (e.g., within fraternity/sorority, residential life, substance use training). While participants provided recommendations relevant to specific student groups (see Table 2), they also recommended campus-wide *advertising* via multiple media channels (email, posters, websites, course syllabi) and in heavily trafficked areas of campus. Many students perceived that it would be effective messaging/framing to communicate that being trained to administer naloxone is part of being a responsible NYC community member (e.g., “global citizen”) and that being trained would provide an opportunity to demonstrate leadership and initiative. A student athlete commented:

I think a lot of students at Columbia are... here to not be followers, but be leaders and so by putting this information out there and framing it as... you’ll be a better leader in whatever community that you go on to after Columbia is probably the best way to frame that.

### Individual characteristics

#### Barriers

Most participants, with the exception of some Military Veterans, Recovery Coalition members, and Columbia Health staff members, said they had little to no interaction with students who use opioids. Most students had limited *knowledge* of and familiarity with naloxone and its ability to reverse opioid overdose. The majority of participants were somewhat aware of the dangers of opioids, specifically fentanyl, and the risk that it could be mixed with other drugs and cause overdose. Students reported perceptions of widespread recreational use of alcohol, marijuana, nicotine, and “study” drugs (e.g., Adderall) on campus, but believed the use of these substances was normalized, while opioid use carried a specific stigma that may prevent students from disclosing use.

A number of students expressed low *self-efficacy* when considering administering naloxone once trained, as well as fear of using naloxone incorrectly or ineffectively. Some student participants also expressed reluctance about assuming responsibility to save a life or feeling obligated to intervene when they see a person who may be overdosing, either on campus or elsewhere in the city (e.g., subway). Some student participants also voiced fear of conduct consequences (e.g., probation, suspension) if they administered naloxone, because they would be “caught” with people who use opioids. RAs generally agreed that it would be prudent for them to carry naloxone, as they are mandated to report instances in which student safety may be compromised. They pointed out that they have limited ability to recognize opioid use and prevent overdose because students are hesitant to disclose their own or their peers’ opioid use for fear of repercussions. One RA shared:

I’ve also heard people talk about how they have some fear of reaching out because they don’t know what’s going to happen… afterward… if there’s going to be any consequences … like if they’re going to follow up … if it’s going to be on their records.

#### Facilitators/recommendations

While opioid use is not considered the most pressing substance use issue on campus, participants agreed that both campus administrators/leadership and students generally have progressive *attitudes* and would be supportive of opioid overdose prevention efforts. Several students said their associations with people who use opioids were “back home” where they knew high school friends or family members who had struggled with opioid use. Participants recommended including messaging/framing around empowering and *motivating* students to get trained to promote safety on campus, in the wider NYC community, and in their hometown communities. They recommended that trainings focus on how to recognize opioid overdose and appropriate circumstances in which to intervene and administer naloxone. A Columbia Health staff member shared:

I would think the administration would support it for all those same reasons, because there is this focus on student safety, on promoting health… so I don’t see why they wouldn’t have tools available to prevent an overdose

### Intervention characteristics

#### Barriers

The majority of student participants asserted that it would be difficult to ensure widespread participation in naloxone trainings due to competing demands for students’ free time (e.g., heavy course loads and extracurricular activities limiting students’ ability to attend trainings). Several students expressed concern that university-sponsored naloxone trainings risked the appearance of condoning opioid use and that some students may be emboldened to use opioids recreationally with the knowledge that their classmates would be able to reverse an overdose. Staff members raised concerns that students’ parents may not support naloxone trainings because of the implication that their children were using opioids. The perceived *complexity* of the training was also noted, as many participants believed it would be difficult to identify which students on campus would most benefit from naloxone training. Further, once trained, participants worried that few people would always carry naloxone or know how to access a kit on campus. As stated by a Fraternity member:

I feel like people don’t really think like ‘my friends are going to overdose on opiates’... So it doesn’t, I don’t think it occurs to them to bother to go get trained or to have Narcan around.

#### Facilitators/recommendations

Students reported that naloxone trainings could be grouped or aligned with pre-existing trainings on related or important topics (e.g., during first-year orientation or other substance use trainings). Students noted that extracurricular affiliations and social media could allow trainings to be advertised within student groups. Participants recommend incentivizing participation in naloxone trainings (e.g., making training extra credit as part of a course or integrating within existing required trainings) and making them conveniently located and timed for students. They suggested including local and national quantitative data on the opioid epidemic to enhance perceived *strength and quality of the evidence* regarding the need for naloxone training. Overall, participants felt that naloxone/opioid trainings should be *adaptable* in that they could be incorporated into existing trainings and incentive structures (e.g., Greek life requirements) and tailored to students’ concerns and interests. Recommendations and priorities for specific student groups for implementation of naloxone/opioid education are presented in Table 2.

## Discussion

Informed by CFIR, this paper seeks to advance understanding of multi-level factors that shape uptake and delivery of naloxone/opioid training on college campuses. While there are a growing number of promising opioid/naloxone training programs being developed and evaluated on college campuses [53–55, 60, 66], particularly within professional graduate education [38, 39, 41], to our knowledge, this is the first theory-informed contextual assessment and evaluation of the key multi-level barriers and facilitators to implementation specifically on an undergraduate college campus.

Through nine focus groups conducted among key stakeholders on an undergraduate college campus, we identified many barriers to implementation of naloxone/opioid training across the five CFIR domains. In the outer setting, stigma surrounding disclosing opioid use and seeking help for substance use issues, fear about legal repercussions of opioid use, and lack of clarification around campus policies (i.e., mandatory reporting, academic/legal concerns) were key concerns. In the inner setting, related to the campus context and implementation processes, key challenges related to the large, decentralized student body, which many participants perceived to impede communication efforts that focused on one-size-fits-all messaging. Additional challenges commonly identified included competing university-wide initiatives/priorities (e.g., sexual assault prevention) and training fatigue, as well as the perception among some students that substance use and mental health resources were not easily accessible.

Related to individual (both students and staff) characteristics, commonly identified barriers to implementation included the perception that other substance use issues were more pressing on campus, and students’ limited knowledge about naloxone and opioids. Several student participants raised concerns about assuming responsibility for others’ lives (once trained) and not having high self-efficacy to respond rapidly and appropriately in various contexts. Related to intervention (e.g., training) characteristics, perceived barriers to implementation included the perceived complexity of the training, competing demands for students’ time due to rigorous academic and extracurricular responsibilities, some concern that increasing naloxone access may encourage opioid use, limited access to kits in buildings and ability to carry kits on person, and challenges in identifying and reaching students with greatest need for training. Though little research has examined barriers to implementing opioid/naloxone education specifically, our findings are consistent and aligned with research on college campuses that has documented some of the common challenges in implementing substance use programming more broadly (e.g., training fatigue, competing demands [61, 67]). It is possible that barriers to implementing opioid/naloxone education may be even more challenging and important to address given some of the perceptions among both staff and students that other substance use issues are higher campus priorities.

Numerous, multi-level facilitators of implementation were identified across CFIR domains. For example, at the outer setting, facilitators included heightened student awareness of the opioid overdose epidemic affecting the wider NYC community and home communities for students not from NYC. Additionally, there were perceptions that students and campus administrators generally have progressive attitudes towards naloxone use and would have the buy-in to prevent opioid overdose (particularly if there were partnerships/resources to supply low-cost/free naloxone). Recommendations to further enhance the reach of the program to students during implementation efforts included providing clarification up front and at the training regarding legal repercussions and mandatory reporting. Additionally, several participants thought buy-in to implementation among campus leadership might be enhanced if training was framed as an opportunity for their institution to be seen as a leader among peer institutions or if there was already high uptake of naloxone training (i.e., an established social norm) at peer institutions.

Additional recommendations to enhance reach and implementation on campus were to directly address some of the barriers and challenges identified above, particularly in terms of how the training should be framed/communicated on campus, thereby shifting the social norm around being trained. Common recommendations included simplifying and standardizing how to access mental health and substance use resources (including opioid/naloxone education) in a centralized way across campus, integrating and aligning incentives for training within existing structures (e.g., aligning with Greek life requirements; integrating into first-year orientation, broader substance use trainings, life-saving trainings like CPR). Additionally, many participants saw value and appeal in framing trainings as important to the broader NYC community and demonstrating leadership and global citizenship across campus. Several participants highlighted the importance of engaging specific campus leaders, “champions,” and credible messengers who were already part of specific social groups (e.g., fraternity leadership for fraternity members) as an important strategy, as well as tailoring the content of the training to students’ specific needs and concerns (e.g., addressing stigma).

To date, little research has investigated how to best support and facilitate widespread and routine implementation of naloxone training and opioid education. Importantly, many of the strategies identified above (e.g., tailored messaging, program champions) align with existing implementation strategies from well-established taxonomies (e.g., Expert Recommendations for Implementing Change taxonomy [68]). Such strategies could be further refined and mapped onto prominent implementation barriers and tested in future research on college campuses to understand their impact on facilitating implementation. Such strategies must be balanced with considerations of resource allocation, supply/access to naloxone, and how to identify and reach those who may most benefit from training [54].

Limitations should be noted. First, our findings sought to provide initial insight into implementation challenges and considerations on college campuses and focused on one university’s experience. We recognize our interviews were conducted at a school that may not reflect all experiences of campuses nationally (e.g., located in a large urban area, private university) and thus, findings are not broadly generalizable to all college campuses (e.g., varying organizational and health-related resources and infrastructure, leadership buy-in, social norms/policies across campuses). Additionally, it would have been ideal to have additional insights from staff/administrators during focus groups; this initial research focuses on a range of student perspectives, with some insight into how this may (not) align with staff perspectives. Future research should seek additional input at the campus staff, faculty, and administrator levels to provide a full range of perspectives. It is important to note that we used CFIR to guide data collection, analysis, and presentation of results here, and in some cases, there was an overlap between where findings best “fit” within CFIR domains; we have presented them here where there was an agreement between coders.

## Conclusions

Despite limitations, to our knowledge this is the first study to provide in-depth insights into potential barriers and facilitators to widespread and routine implementation of naloxone/opioid education on undergraduate college campuses. The study captured a diverse range of student groups and staff perspectives and was grounded in an implementation science framework to enhance the rigor and communication of findings. Notably, we found that while CFIR was developed and has been commonly applied in healthcare, application of CFIR to inform implementation on a college campus setting was feasible, though required explicit specification of codes during analysis. For example, in some cases, we needed to refine or expand upon specific CFIR constructs or domains in our coding or specify if factors were barriers or facilitators. As another example, we added codes for stigma, given that it was an important barrier to attending training or using naloxone (in both the Outer Context and Implementer Characteristics).

This research contributes to the growing literature on the application and refinement of CFIR across diverse community contexts [69–71]. Given the large number of CFIR constructs, our findings indicate that to inform the next steps in tailoring naloxone training for implementation on college campuses, it might be important to first prioritize understanding key barriers and facilitators in the inner (campus) context. This includes assessing what existing processes, incentive structures, organizational resources, and programs are available on campus with which training could be aligned. It may also be useful to prioritize the assessment of existing champions and communication channels that could be leveraged and tailored to reach priority campus groups. Finally, it might be important to assess leadership support of naloxone training (and naloxone costs/availability), since that will have implications for being able to widely implement and ultimately sustain the program.

## Supplementary Information

**Additional file 1.** Focus Group Questions. This is the semi-structured, in-depth group guide used by the moderators of all 9 focus groups.

**Additional file 2.** Consolidated criteria for reporting qualitative research (COREQ): 32-item checklist. This checklist meets the reporting standard for qualitative research.

## Data Availability

The datasets generated and/or analyzed during the current study are not publicly available to protect participant confidentiality (e.g., publishing focus group transcripts would violate participant confidentiality, regardless of redaction) but additional illustrative quotes are available from the corresponding author on reasonable request.

## Abbreviations

CDC: Centers for Disease Control and Prevention
CFIR: Consolidated Framework for Implementation Research
IMF: Illicitly manufactured fentanyl
NYC: New York City
OUD: Opioid use disorder
RA: Resident Assistant

## Footnotes

1 Good Samaritan laws enable people to call public safety / 911 without fear of arrest if they require emergency medical services due to themselves having an overdose and/or they witness someone else having an overdose.
