# Supplementary material for: Application of The Consolidated Framework for Implementation Research to inform understanding of barriers and facilitators to the implementation of opioid and naloxone training on college campuses
Source: Implement Sci Commun. 2023 May 23;4:56. doi: 10.1186/s43058-023-00438-y (PMC10204023; doi:10.1186/s43058-023-00438-y)
Supplement: Supplementary file 1 — Additional file 1. Focus Group Questions. This is the semi-structured, in-depth group guide used by the moderators of all 9 focus groups. [file 43058_2023_438_MOESM1_ESM.docx]

**Focus Group Questions**

**Introduction:**

*Moderator:*

*Hi everyone, and thank you for being here today to share your thoughts, opinions, and experiences with us.*

*We are interested in learning more about your perceptions and attitudes towards opioid use, opioid associated resources, and opioid overdose reversal. We’re also interested in learning about the needs of Columbia undergraduate students with respect to these issues on the Morningside campus, and what you see may be barriers or facilitators to implementing opioid education and opioid overdose reversal training on campus. As undergraduate students at Columbia, you are the experts on what Columbia undergraduates need.*

*Before we get started, I want to talk about logistics and set a few “ground rules” for the session:*

- *This session will go for one hour*
- *Bathrooms are located….*
- *Only one person talks at a time*
- *While we will be recording the session for transcribing purposes, please know: Confidentially is assured - Information that is shared in this group will not result in any academic or administrative repercussions. It is important for us to learn about student perspectives so that we can work to improve the student experience at Columbia University.*
- *It is important for us to hear everyone’s ideas and opinions. There are no right or wrong answers to questions – all sentiments, suggestions, and experiences are valuable.*
- *It is important for us to hear all sides of an issue – both positive and negative. So while we ask that you share responsibly and with respect, we also invite you to do so honestly and openly. We want to know what you really think.*

*Does anyone have any questions at this point?*

**Icebreaker:**

*Moderator:*

*To get started, we’re going to do a quick introduction activity and will need you to provide verbal consent to be recorded during this session.*

*Please share your:*

- *Name, pronouns, school, year in school, group you are representing (specific sports team, fraternity, or branch of military), and tell us about some of your interests on campus (e.g., organized social groups, use of social media, clubs you are a member of, etc.)*
- *Verbal consent to be recorded*

*I’ll go first:*

*Name: ______ ; pronouns_______; school__________; year__________; and some of my interests on campus are: ____________. I consent to be recorded.*

**Question 1: Drug use and opioid use on college campuses**

*Moderator:* *the first set of questions will be about substance use. This subject may be sensitive but any information that we gain in this focus group will assist us in our research.*

- When you think of “drug use” on a college campus, what comes to mind?
  - Probe: Of these drugs, which are of the most concern to you on the Columbia campus? To members of your community?
- When you think of “opioids”, what comes to mind?
- Describe the factors that you think influence the risk of opioid use among students at Columbia University/within your community.
  - *Probes: Locations (e.g., living situation or social situations, stress, peers, social media, family/personal struggles, academic expectations/pressure, loneliness)?*
  - *Probe: Use of multiple substances?*
  - *Probe: What have you heard about fentanyl in relation to opioids or drug use?*
- Describe the ways that you think students may try to protect themselves or each other from the risk of opioid use/misuse.

**Transition Question:**

- How have you or members of your community been influenced by opioid use?

Question 2. **Opioid overdose reversal/ naloxone/ Narcan**

- What do you know about opioid reversal drugs?

*Moderator: the next set of questions will discuss naloxone/Narcan. Naloxone, also known as Narcan, is an opioid-reversal drug that can be administered by a medical professional or any layperson who is appropriately trained. The medication can be administered through the nose/ intranasally.*

- What have you heard, or do you know about naloxone, also known as Narcan?

**Question 3. Opioid education and opioid overdose reversal training at Columbia University**

- What resources are currently available at Columbia University to assist students with opioid / substance use?
  - Are there barriers that you’re aware of for students to access those? If so, what are they?
- How do you feel about the possibility of opioid/naloxone training being provided at Columbia?
  - *How well would it fit with the values and norms of your community [or as a member of the student body]?*
  - *How do you think your friends and family will perceive your participation in a naloxone training program?*
  - *What are objections you may have to an education and training program?*
- What do you feel is the general level of receptivity at Columbia University to providing student education around opioids and training to administer opioid reversal drugs like naloxone among students? Why?
- *Your peers and community?*
- *The administration?*
- *Student leadership?*
- Do you think making education and training related to opioid and naloxone available should be a priority at Columbia University?
- Which medium for learning about opioid education and naloxone do you think would be the most accessible and appealing to students (e.g., online module, in-person training, video, fact sheets, etc.)? What are your recommendations for delivering trainings to reach a range of students across campus? Are there any key facilitators that would help support delivery of these trainings?
- If a naloxone training were available to students on campus, what do you think are some of the key barriers students might face to attending the educational sessions/trainings?
  - What are some facilitators? (probe for factors at the policy, organizational, implementation, intervention, staff/student levels)
- After attending an opioid education session and naloxone education and training, what are some barriers you might face to using the information learned in the trainings?
  - What are some facilitators? (probe for factors at the policy, organizational, implementation, intervention, staff/student levels)

**Wrap-Up Question:**

- What specifically would you like to see Columbia University doing in relation to opioid use on or around campus?

**Ending Question:**

- What else would you like to share about opioid use or opioid prevention at Columbia or in your community?

*Thank you very much for your time today.*
