# Supplementary material for: Application of The Consolidated Framework for Implementation Research to inform understanding of barriers and facilitators to the implementation of opioid and naloxone training on college campuses
Source: Implement Sci Commun. 2023 May 23;4:56. doi: 10.1186/s43058-023-00438-y (PMC10204023; doi:10.1186/s43058-023-00438-y)
Supplement: Supplementary file 2 — Additional file 2. Consolidated criteria for reporting qualitative research (COREQ): 32-item checklist. This checklist meets the reporting standard for qualitative research. [file 43058_2023_438_MOESM2_ESM.docx]

For manuscript titled “Application of the Consolidated Framework for Implementation Research to Advance Understanding of Barriers & Facilitators to the Implementation of Naloxone Training on College Campuses”

Consolidated criteria for reporting qualitative research (COREQ): 32-item checklist

| **No** | **Item** | **Guide questions** | **Page #/Description** |
| --- | --- | --- | --- |
| **Domain 1: Research team and reflexivity** | | | |
| Personal Characteristics | | | |
| 1. | Interviewer/ facilitator | Which author/s conducted the interview or focus group? | Done – Pages 4 and 13 |
| 2. | Credentials | What were the researcher’s credentials? E.g. PhD, MD | Not included in manuscript:   1. Rachel C. Shelton, ScD, MPH 2. Kathleen Goodwin, MDc 3. Michael McNeil, EdD, CHES, FACHA 4. Melanie Bernitz, MD, MPH 5. Savannah Alexander, MPH 6. Carrigan Parish, PhD, DDS 7. Laura Brotzman, MPH 8. Matthew Lee, DrPH, MPH 9. WaiKwan (Bonnie) Li, MA 10. Supriya Makam, BAc 11. Nicholas Ganek, BAc 12. Dean Foskett, BAc 13. Chloe Warren, MPHc 14. Lisa R. Metsch, PhD |
| 3. | Occupation | What was their occupation at the time of the study? | Done – Page 14  Not included in manuscript:  Rachel C. Shelton   - Associate Professor, Columbia University - Deputy Chair for Faculty Development and Research Strategy - Mailman School of Public Health, Dept. of Sociomedical Sciences - Co-Director of Community Engagement Core Resource - Director, Implementation Science Initiative - Columbia's Irving Institute for Clinical and Translational Research   Kathleen Goodwin   - MD Candidate - Columbia University Vagelos College of Physicians and Surgeons   Michael McNeil   - Chief of Administration, Columbia Health - Adjunct Assistant Professor, Sociomedical Sciences - Mailman School of Public Health - Columbia University   Melanie Bernitz   - Associate Vice President and Medical Director, Columbia Health - Associate Clinical Professor of Medicine (in the Center for Family and Community Medicine)   Savannah Alexander   - Project Coordinator, Columbia University - Mailman School of Public Health, Department of Sociomedical Sciences   Carrigan Parish   - Assistant Professor, Columbia University - Mailman School of Public Health, Dept. of Sociomedical Sciences   Laura Brotzman   - Project Coordinator, Columbia University - Mailman School of Public Health, Department of Sociomedical Sciences   Matthew Lee   - Assistant Professor - Department of Population Health, Section for Health Equity - NYU Grossman School of Medicine - NYU Langone Health   WaiKwan (Bonnie) Li   - Research and Quality Manager - Columbia Health   Supriya Makam   - BA student, Neuroscience and Behavior - Columbia University   Nicholas Ganek   - BA student - Columbia University   Dean Foskett   - BA student - Columbia University   Chloe Warren   - MPH student - Columbia University Mailman School of Public Health   Lisa R. Metsch   - Dean, Columbia School of General Studies - Professor of Sociomedical Sciences - Columbia University |
| 4. | Gender | Was the researcher male or female? | Not included in manuscript:   1. Rachel C. Shelton - female 2. Kathleen Goodwin - female 3. Michael McNeil - male 4. Melanie Bernitz - female 5. Savannah Alexander - female 6. Carrigan Parish - female 7. Laura Brotzman - female 8. Matthew Lee - male 9. WaiKwan (Bonnie) Li - female 10. Supriya Makam - female 11. Nicholas Ganek - male 12. Dean Foskett - male 13. Chloe Warren - female 14. Lisa R. Metsch - female |
| 5. | Experience and training | What experience or training did the researcher have? | Done – Page 4  Not included in manuscript:   1. Rachel C. Shelton – Dr. Shelton is a social and behavioral scientist with expertise in implementation science, sustainability, health equity, and community-based participatory research. She has 15 years of experience conducting mixed-methods research in both community-based and health system settings, with over 100 peer-reviewed publications in journals including Translational Behavioral Medicine, Social Science & Medicine, American Journal of Public Health, Implementation Science, and Annual Reviews of Public Health. 2. Kathleen Goodwin – Ms. Goodwin has training in quantitative and qualitative analysis and as a physician. 3. Michael McNeil – Dr. McNeil brings expertise in education and training, health promotion, evaluation, and college student health. 4. Melanie Bernitz – Dr. Bernitz brings expertise in clinical and community medicine, health promotion, and college student health. 5. Savannah Alexander – Ms. Alexander has expertise in qualitative research methods and the field of mental health and substance use. 6. Carrigan Parish – Dr. Parish brings expertise in quantitative research and substance use. 7. Laura Brotzman – Ms. Brotzman has expertise in mixed-methods research and implementation science. 8. Matthew Lee – Dr. Lee has expertise in mixed-methods research, community-based participatory research, implementation science, sustainability, and health equity. 9. WaiKwan (Bonnie) Li – Ms. Li has expertise in health promotion and college student health. 10. Supriya Makam – Ms. Makam has lived experience as an undergraduate student at Columbia University and has training and experience in qualitative collection and opioid/substance use research. 11. Nicholas Ganek – Mr. Ganek has lived experience as an undergraduate student at Columbia University and has training and experience in qualitative collection and opioid/substance use research. 12. Dean Foskett – Mr. Foskett has lived experience as an undergraduate student at Columbia University and has training and experience in qualitative collection and opioid/substance use research. 13. Chloe Warren – Ms. Warren has expertise in health promotion and research methods for conducting and analyzing quantitative and qualitative data in public health. She also has expertise in implementation science. 14. Lisa R. Metsch – Dr. Metsch brings expertise in intervention development, community intervention trials and substance use. |
| Relationship with participants | | | |
| 6. | Relationship established | Was a relationship established prior to study commencement? | Not included in manuscript:   - A relationship was not established with participants prior to study commencement. |
| 7. | Participant knowledge of the interviewer | What did the participants know about the researcher? e.g. personal  goals, reasons for doing the research | Done – Additional file 1  Not included in manuscript:   - All focus group participants were provided with an information sheet as part of the informed consent process, which detailed the research purpose; study abstract; research questions and aims; risks, benefits, and alternatives to participation; confidentiality safeguards; compensation; voluntary nature of study participation; and statement of consent. |
| 8. | Interviewer characteristics | What characteristics were reported about the inter viewer/facilitator?  e.g. Bias, assumptions, reasons and interests in the research topic | Done – Additional file 1  Not included in manuscript:   - The information sheet described above outlined the research purpose, conveying the researchers’ interests in the research topic. |
| **Domain 2: study design** | | | |
| Theoretical framework | | | |
| 9. | Methodological orientation and theory | What methodological orientation was stated to underpin the study? e.g. grounded theory, discourse analysis, ethnography, phenomenology,  content analysis | Done – Pages 1 and 4 |
| Participant selection | | | |
| 10. | Sampling | How were participants selected? e.g. purposive, convenience,  consecutive, snowball | Done – Pages 3-4 |
| 11. | Method of approach | How were participants approached? e.g. face-to-face, telephone, mail,  email | Not included in manuscript:   - As part of the environmental scan referenced in the paper, emails were sent out to student organization leaders and Columbia University staff, inviting them to focus groups. - Recruitment occurred person to person and via email. |
| 12. | Sample size | How many participants were in the study? | Done – Page 5 |
| 13. | Non-participation | How many people refused to participate or dropped out? Reasons? | We did not collect this information, and it is infeasible to determine. |
| Setting | | | |
| 14. | Setting of data collection | Where was the data collected? e.g. home, clinic, workplace | Not included in manuscript:   - The focus groups were conducted in a neutral, non-formal or academic study-focused space and setting at Columbia University that allowed for the participants to relax and speak freely in response to the questions asked. |
| 15. | Presence of non-participants | Was anyone else present besides the participants and researchers? | Not included in manuscript:   - No one besides the participants and researchers were present during the focus groups. |
| 16. | Description of sample | What are the important characteristics of the sample? e.g. demographic  data, date | Done – Pages 3-5; Table 1 |
| Data collection | | | |
| 17. | Interview guide | Were questions, prompts, guides provided by the authors? Was it pilot  tested? | Done – Additional file 1  Not included in manuscript:   - We pilot tested the focus group guide and refined it after the first focus group. |
| 18. | Repeat interviews | Were repeat inter views carried out? If yes, how many? | Not included in manuscript:   - No repeat interviews or focus groups were carried out. |
| 19. | Audio/visual recording | Did the research use audio or visual recording to collect the data? | Done – Page 4 |
| 20. | Field notes | Were ﬁeld notes made during and/or after the interview or focus group? | Not included in manuscript:   - Field notes were made after each focus group. |
| 21. | Duration | What was the duration of the inter views or focus group? | Not included in manuscript:   - Focus group duration ranged from approximately 40 minutes to 1 hour and 20 minutes. |
| 22. | Data saturation | Was data saturation discussed? | Done – Page 5 |
| 23. | Transcripts returned | Were transcripts returned to participants for comment and/or correction? | Not included in manuscript:   - Transcripts were not returned to participants for comment and/or correction. |
| **Domain 3: analysis and findings** | | | |
| Data analysis | | | |
| 24. | Number of data coders | How many data coders coded the data? | Done – Page 4 |
| 25. | Description of the coding tree | Did authors provide a description of the coding tree? | Done – Pages 4-5, Figure 1 |
| 26. | Derivation of themes | Were themes identiﬁed in advance or derived from the data? | Done – Pages 4-5, Figure 1 |
| 27. | Software | What software, if applicable, was used to manage the data? | Not included in manuscript:   - We did not use software. We completed qualitative data analysis by hand and using Word documents. |
| 28. | Participant checking | Did participants provide feedback on the ﬁndings? | Not included in manuscript:   - Participants did not provide feedback on the findings. |
| Reporting | | | |
| 29. | Quotations presented | Were participant quotations presented to illustrate the themes/ﬁndings?  Was each quotation identiﬁed? e.g. participant number | Done – Pages 6-11; Tables 3 and 4 |
| 30. | Data and findings consistent | Was there consistency between the data presented and the ﬁndings? | Done – Pages 6-11; Tables 3 and 4 |
| 31. | Clarity of major themes | Were major themes clearly presented in the ﬁndings? | Done – Pages 6-11; Tables 3 and 4 |
| 32. | Clarity of minor themes | Is there a description of diverse cases or discussion of minor themes? | Done – Pages 6-11; Tables 3 and 4 |
